# Mesoporous activated carbon from *Tabebuia aurea* leaves for effective adsorptive removal of 2,4-dichlorophenoxyacetic acid

**DOI:** 10.1038/s41598-025-01647-2

**Published:** 2025-05-16

**Authors:** H. Sridevi, Ramesh Vinayagam, Gokulakrishnan Murugesan, Raja Selvaraj

**Affiliations:** 1 Department of Civil Engineering, Manipal Academy of Higher Education, Manipal Institute of Technology, Manipal, Karnataka 576104 India; 2 Department of Chemical Engineering, Manipal Academy of Higher Education, Manipal Institute of Technology, Manipal, Karnataka 576104 India; 3 Department of Biotechnology, M.S.Ramaiah Institute of Technology, Bengaluru, Karnataka 560054 India

**Keywords:** *Tabebuia aurea*, Activated carbon, Adsorption, 2,4-dichlorophenoxyacetic acid, Wastewater treatment, Environmental sciences, Materials science, Chemical engineering, Chemical engineering, Environmental chemistry, Materials chemistry

## Abstract

The extensive use of herbicides in agriculture contributes to water pollution, posing a significant environmental risk. This study focused on the H_3_PO_4_ activation of *Tabebuia aurea* leaves to prepare activated carbon. The resulting adsorbent was then tested for its capability to eliminate 2,4-dichlorophenoxyacetic acid (2,4-D), the heinous herbicide. The prepared activated carbon demonstrated a mesoporous nature with a high specific surface area (773.21 m^2^/g). Moreover, the existence of carbon and oxygen was proven using elemental analysis, and a post-adsorption chlorine signal confirmed 2,4-D presence. It was found that pH 2 provided optimal adsorption conditions, with an activated carbon dose of 0.45 g/L and 50 mg/L 2,4-D concentration. The process comprehended pseudo-second-order kinetics, as determined by kinetic modeling. The Langmuir isotherm model described the adsorption equilibrium, indicating a maximum adsorption capacity of 158.28 mg/g. Thermodynamic tests indicated physisorption and exothermicity. The prepared activated carbon also demonstrated substantial regeneration capacity up to five cycles. Consistent adsorption capacity proves its efficacy across diverse water matrices. Adsorption efficiency remained largely unaffected by inorganic ions at 0.1 and 1 mM concentrations. This confirms the viability of the synthesized adsorbents and their affordable use for 2,4-D removal in water remediation.

## Introduction

Water pollution is a pressing crisis requiring immediate attention. Its impact is widespread, jeopardizing both human and environmental health, causing significant economic damage, and exacerbating water scarcity. A key culprit is agricultural runoff, which washes pollutants like fertilizers and pesticides from farms into the rivers and other water sources. Over time, the application of crop protection products has grown substantially and it’s estimated that pests – insects, weeds, mites, rodents, and nematodes – are responsible for a staggering 45% of annual food production losses^1^. Herbicides are agents utilized for the management of unwanted vegetation. Phenoxy herbicides are the most widely used type of herbicide, and among these, 2,4-Dichlorophenoxyacetic acid (2,4-D) is prominent owing to its efficacy and affordability^2^. It’s an herbicide that mimics auxin, a plant hormone and is used to control broadleaf weeds. It is highly water-soluble, has low biodegradability, and is weakly absorbed by soil, making it a potential contaminant in water bodies, which can seriously impact aquatic environments. It is prevalent in urban rivers, primarily resulting from residential applications^3^. Its presence in aquatic ecosystems is concerning due to its carcinogenic nature, making the elimination and breakdown of 2,4-D from water sources a critical priority.

Adsorption is preferred over other techniques to remove 2,4-D, owing to its effectiveness, affordability, and user-friendliness. In addition, it efficiently removes herbicides, even at low concentrations, and prevents the generation of byproducts. Pesticides are routinely removed from wastewater using adsorbents such as clay and activated carbon^4^. Due to the several drawbacks of commercial activated carbon, including its expensive cost and the non-renewable nature of coal-based starting materials, research has switched to investigating more affordable adsorbents produced from waste materials, including agricultural byproducts^5^. Studies have demonstrated the effective 2,4-D removal by employing activated carbon sourced from agricultural residues, such as *Retama-monosparma* (L.) Boiss^6^, *Syagrus romanzoffiana*^7^ and date palm coir waste^8^. Although biomass-derived activated carbon is readily available, its primarily microporous structure restricts the adsorption of larger molecules. Consequently, research has focused on developing mesoporous activated carbon, characterized by pore sizes ranging between 2 and 50 nm. Mesoporous carbon offers numerous benefits, including tailored pore size, substantial specific surface area, abundant pore volumes, good mechanical stability, exceptional electrical conductivity, and minimal cost, making it a significantly better adsorbent^9^. Furthermore, research on using mesoporous-activated carbon specifically for 2,4-D adsorption is surprisingly limited.

Synthesis of activated carbon can be done via physical or chemical activation processes. Activation and carbonization occur simultaneously in chemical activation when different dehydration agents including H_2_SO_4_, ZnCl_2_, KOH, and H_3_PO_4_ are used^10^. Chemical activation, a key method for developing porous structures, involves thermally treating a biomass feedstock mixed with an activating agent. This process facilitates pore formation through reactions like condensation, dehydration, and aromatization^11^. Although several activating agents are used in activated carbon production, H_3_PO_4_ is often preferred due to its relatively safer handling and more environmentally benign^12^. H_3_PO_4_ reacts with biopolymers, creating cross-links that connect the resulting fragments into new polymeric phosphate structures via phosphate ester linkages. This prevents pore shrinkage and collapse at high temperatures. Additionally, H_3_PO_4_ activation promotes mesoporous structure development by forming “phosphoric acid-polymer” complexes at elevated temperatures^13^. This unique, robust, mesoporous structure, along with improved thermal stability, makes the resulting activated carbon highly effective at removing pollutants from wastewater. Recently, several studies synthesized activated carbon using H_3_PO_4_ as an activating agent from various biomass such as cocoa pod^14^, S*pathodea campanulata* flowers^15^, teak sawdust^16^, and coffee husk^17^. This research aimed to produce activated carbon from *Tabebuia aurea* leaves using H_3_PO_4_ as an activating agent. *T.aurea*, also recognized as the silver trumpet or Caribbean trumpet tree, is a deciduous ornamental tree with vibrant yellow flowers, commonly found in tropical and subtropical regions^18^. The substantial seasonal leaf litter from this widely cultivated ornamental and shade tree provides a significant resource for activated carbon synthesis. Utilizing these *T. aurea* leaves not only valorizes waste but also supports sustainable material development. As with typical plant biomass, the *T.aurea* leaves are also rich in lignocellulosic and phytochemical components result in high carbon yields and porous structures upon carbonisation^19,20^. This relatively unexplored biomass holds promising potential as a novel, cost-effective, and eco-friendly alternative for adsorbent production compared to more conventional agricultural waste materials.

This article describes the process of preparing mesoporous activated carbon with *T. aurea* leaves using H_3_PO_4_. The synthesized adsorbent was characterized, and its potential to adsorb 2,4-D was assessed by considering variables including pH, temperature, dosage, and 2,4-D concentration. The adsorption dataset was assessed using kinetic, thermodynamic equations, and isotherm models. Moreover, the reusability potential of the adsorbent and the efficacy in the existence of various inorganic ions were investigated.

## Materials and methods

### Materials

All the chemicals used here were of analytical grade. Sisco supplied the 2,4-D. Merck, India provided H_3_PO_4_ (85% purity), sodium bicarbonate and NaOH. Leaves of *T. aurea* were collected from trees on the Manipal Campus, India.

### Synthesis of activated carbon

**Fig. 1:**
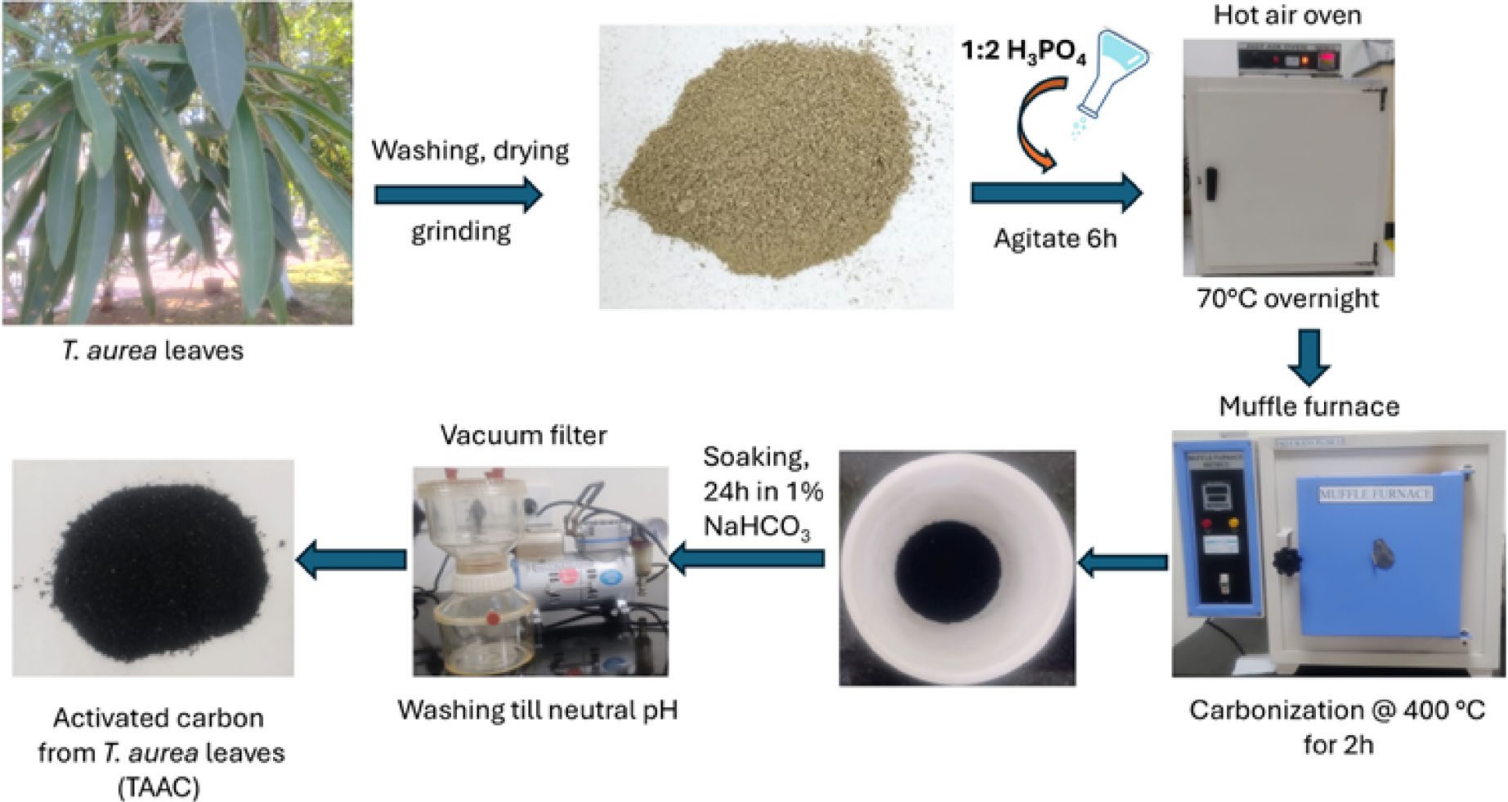
Synthesis of TAAC from *T.aurea* leaves by H_3_PO_4_ activation.

*T. aurea* leaves were collected, cleaned, oven-dried, and pulverized. The resulting powder was stored for later use. Five grams of this powder were mixed with H_3_PO_4_ (1:2 ratio) and agitated for six hours. The contents were then dehydrated overnight by incubating at 70 °C in hot air oven, followed by carbonization at 400 °C for 2 h in a muffle furnace. The material produced was soaked in a 1% sodium bicarbonate solution for 24 h, rinsed up to a neutral pH, and then desiccated by subjecting to 100 °C for 24 h (Fig. 1). This *T. aurea* leaves-derived activated carbon, labeled as TAAC, was stored in an airtight container.

### Characterization methods

A SHIMADZU-8400 S equipment was used to acquire the FTIR spectra of the adsorbent. XRD study was conducted using a Japanese-made Rigaku Miniflex 600 X-ray diffractometer. Using a field emission scanning electron microscopy (FESEM) system developed by German-made Carl ZEISS SIGMA instrument, morphological characteristics were investigated. An American-made PHI 5000 Versa Probe III XPS equipment was used for the analysis of elemental composition. Measurements of pore volume and specific surface area were conducted by gas sorption analysis using a Brunauer–Emmett–Teller apparatus Smart Instruments, Mumbai.

### Batch adsorption tests

Experimental batch trials were done using 100 mL of aqueous solutions at 303 K, changing variables including pH, contact time, TAAC dosage, and 2,4-D concentration with constant agitation of the solutions using an incubator shaker. 2,4-D concentration was analyzed by employing a UV-visible spectrophotometer (UV1900, SHIMADZU, Japan) at a wavelength of 283 nm after the adsorbent was removed. Equations (1) and (2) were employed to evaluate the efficiency of 2,4-D removal and equilibrium adsorption capacity (q_e_, mg/g) respectively.

1 $$ 2,4{\text{-D~removal~}}\left( {\text{\% }} \right) = \frac{{\left( {{\text{C}}_{0}  - {\text{~C}}_{{\text{f}}} } \right)}}{{{\text{C}}_{0} }}~ \times 100 $$

2 $$\:\text{q}\text{e}=\frac{\left({\text{C}}_{0}-\:{\text{C}}_{\text{e}}\right)}{m}\:\text{V}$$

The 2,4-D concentrations measured in mg/L at the initial, final, and equilibrium phases are represented here by C_0_, C_f_, and C_e_, respectively. The weight of the TAAC is denoted by m (g), and the volume is symbolized as V (L).

### Adsorption models

Adsorption kinetics were examined by quantifying the uptake of 2,4-D over time, concurrently sustaining other experimental parameters invariable at their optimized levels. Non-linear regression analysis was implemented to assess the fitness of the empirical data to pseudo-first-order (PFO), and pseudo-second-order (PSO) kinetic models Eqs. (3), (4).

3 $$\:{q}_{t}=\:{q}_{e}\:(\:1-\:{e}^{{-k}_{1}t})$$

4 $$\:{q}_{t}=\:\frac{{q}_{e}^{2}\:{k}_{2}\:t}{1+\:{q}_{e}\:{k}_{2}\:t}$$

wherein, q_t_ and q_e_ express the respective adsorption capacities in mg/g at specified time ‘t’ and at equilibrium in minutes. The variable k_1_ (min^− 1^) signifies the PFO constant, while k_2_ (g/ mg. min) represents the PSO constant.

The 2,4-D adsorption data was analyzed by applying three isotherm models: Freundlich, Langmuir, and Jovanovic Eqs. (5)–(7). These isotherm models were employed with the experimental data developed from batch trials conducted under optimized conditions.

5 $$\:{q}_{e}=\:{K}_{F}{C}_{e}^{1/n}$$

6 $$\:{q}_{e}=\:\frac{{q}_{m}\:{K}_{L}{C}_{e}}{\left(1+{K}_{L}{C}_{e}\right)}$$

7 $$\:{q}_{e}={q}_{m}(1-\text{exp}(-{K}_{j}{C}_{e}))$$

wherein, q_m_ indicates the maximum uptake measured in mg/g. K_L_ signifies the Langmuir constant, with units of L/mg. The term 1/n represents the exponent in the Freundlich isotherm, while K_F_ is the Freundlich constant, measured in (mg/g) (mg/L)^−1/n^. K_j_ refers to the Jovanovic constant, expressed in L/mg.

To determine the relationship amongst temperature and 2,4-D adsorption, equilibrium tests were performed across the temperatures ranging from 293 to 323 K, however holding all other parameters at their optimized values. Thermodynamic parameters, specifically the standard entropy change (ΔS°) and standard enthalpy change (ΔH°) were established via a non-linear Van’t Hoff model Eq. (8). The standard Gibbs free energy (ΔG°) was subsequently computed using Eq. (9).

8 $$\:{K}_{T}=\text{exp}\left[\left(\frac{\varDelta\:S^\circ\:}{R}\right)-\left(\frac{\varDelta\:H^\circ\:}{R}\right)\frac{1}{T}\right]$$

9 $${\text{DG}}^\circ =-{\text{RT ln }}{{\text{K}}_{\text{T}}}$$

where, K_T_ (= q_e_/C_e_) is the distribution coefficient.

## Results and discussions

### Characterization results

#### Surface structure and elemental characterization

**Fig. 2:**
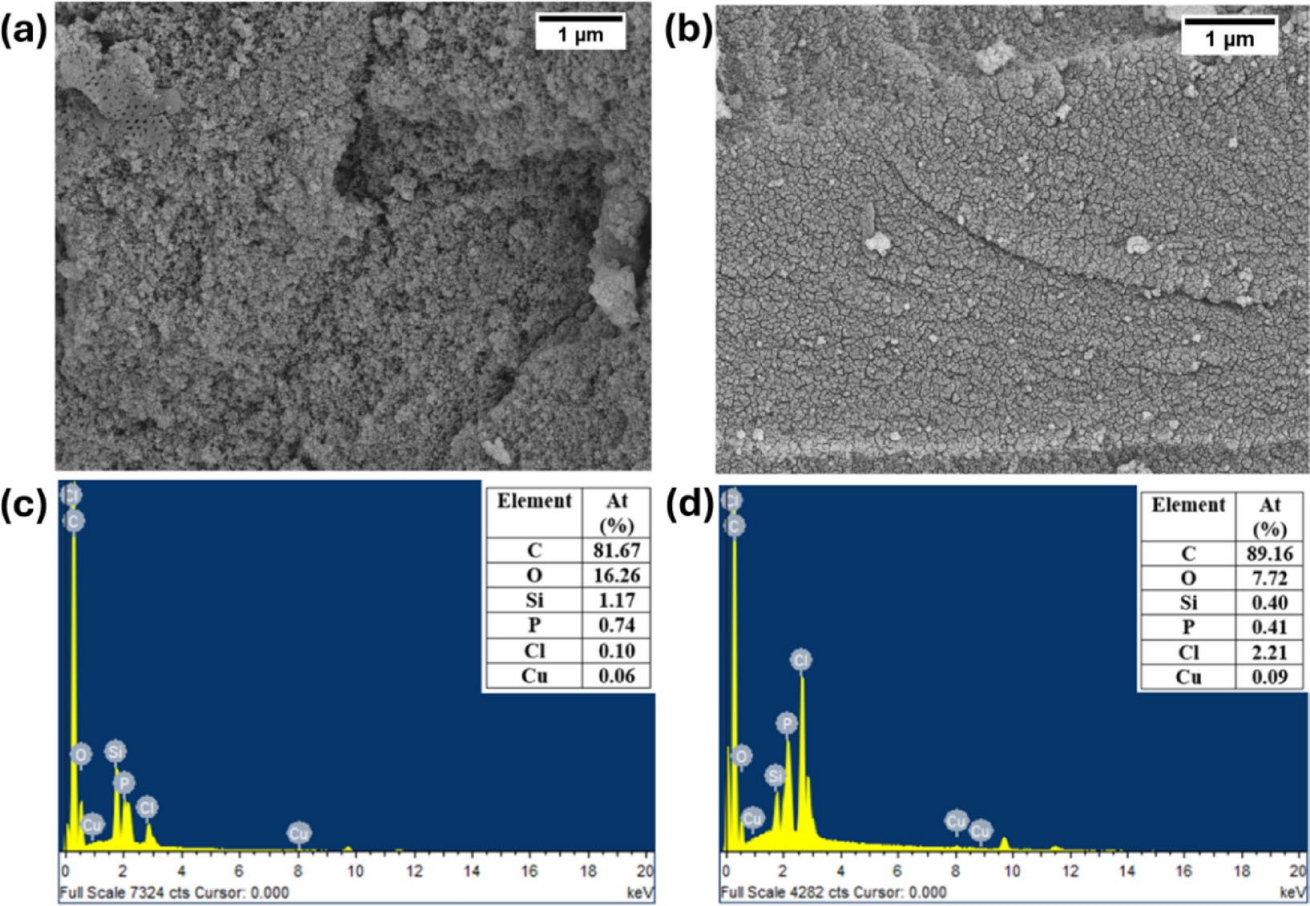
FESEM image (**a**) pre- adsorption (**b**) post-adsorption. EDS image (**c**) pre- adsorption (**d**) post-adsorption.

The surface morphology of the synthesized TAAC is shown in the FESEM image (Fig. 2a), which highlights a porous structure with an irregular shape. The irregularly spaced pores were a sign of H_3_PO_4_ activation, which encourages the growth of a vast surface area^21^. Many other studies also reported similar patterns for activated carbon synthesized using H_3_PO_4_ as an activating agent such as pomegranate pomace^22^ and walnut shells^23^. However, as 2,4-D was adsorbed, the TAAC’s morphological and surface characteristics changed, which caused the molecules’ pores to fill with 2,4-D, forming aggregates as witnessed in (Fig. 2b). BET analysis revealed that the synthesized TAAC has a pore volume of 0.7767 cm³/g with a specific surface area (SSA) of 773.21 m²/g and pore diameter of 3.99 nm. As the average pore diameter falls within the mesoporous range (2–50 nm), the synthesized TAAC may exhibit mesoporous characteristics. Few studies have utilized similar mesoporous activated carbons for 2,4-D removal such as date palm coir waste-derived activated carbon with a pore diameter of 2.28 nm^8^, canola stalk-derived activated carbon exhibiting pores of 4.1 nm^24^, and activated carbon from southern yellow pine with a pore size of 4.43 nm^25^. Furthermore, the SSA observed in this work is substantially greater than reported, such as H_3_PO_4_ activated carbon from *Tamarix gallica* leaves with an SSA of 580.37 m^2^/g^26^, corn straw which has an SSA of 463.89 m²/g^27^, and Raffia palm shells with 456.10 m^2^/g^28^.

EDS analysis (Fig. 2c) confirmed the successful synthesis of activated carbon and its elemental composition. The analysis revealed the presence of carbon (81.67%), oxygen (16.26%), phosphorus (0.74%), and trace amounts of other elements. Characteristic peaks were observed for carbon at 0.27 keV, oxygen at 0.5 keV, and phosphorus at 2.0 keV, the latter likely originating from the H_3_PO_4_ used during activation. Small peaks at 2.621 keV (chlorine) may be attributed to the intrinsic composition of the *T. aurea* leaf^14^. A peak at 0.739 keV (silicon) is likely due to the use of glass specimens during analysis. The gold peak at 9.712 keV is an artifact of the gold sputtering process used to enhance image quality. Following 2,4-D adsorption (Fig. 2d), the carbon content increased to 89.16%, with the chlorine content of 2.21%, suggesting chlorine adsorption onto the TAAC. This increase in chlorine content is also reflected in the increased intensity of the Cl peak.

#### XRD, FTIR, and XPS results

**Fig. 3:**
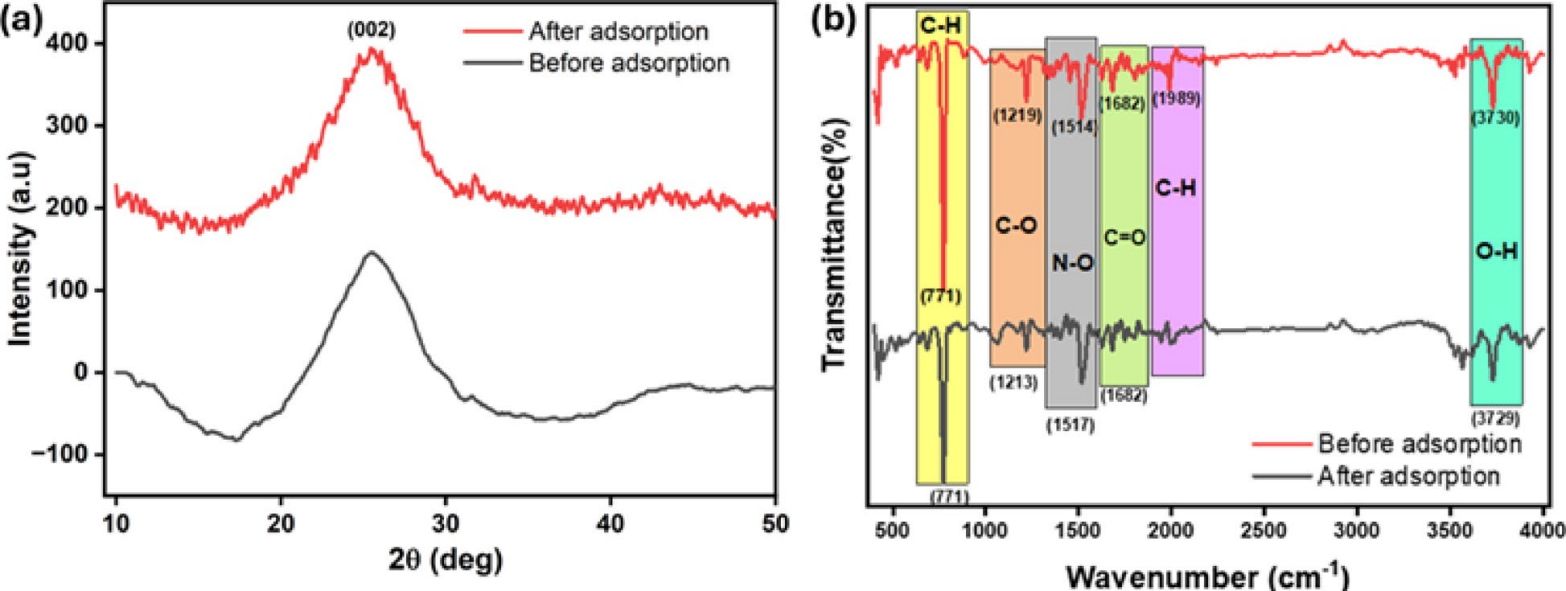
(**a**) XRD image (**b**) FTIR spectra.

The existence of amorphous carbon was confirmed by the large diffraction band between 17 and 30° of XRD spectra. Figure 3a shows a disordered lattice structure indicated by the (002) plane peak at 25.51°. H_3_PO_4_ activation increases disorder and alters interlayer spacing by incorporating phosphorus into the carbon matrix^12^. Following 2,4-D adsorption, XRD analysis revealed significant spectral changes. The (002) plane peak shifted slightly to 25.42° with a decrease in intensity, indicating reduced structural order. This intensity reduction suggests diminished graphitic content, probably caused by 2,4-D binding to carbon functional groups, disrupting the carbon layer arrangement. These XRD changes highlight the impact of 2,4-D adsorption on the TAAC, indicating associations with the carbon matrix, potentially through complex formation or active site occupation^29^.

Figure 3b exhibits the FTIR spectra pre- and post-adsorption. Before adsorption, a weak band with a weak signal was witnessed around 3729 cm^− 1^, assigned to free O-H functional groups of polyphenolic compounds^30^. An insignificant spike at 1989 cm^− 1^ is linked to the C-H bending of aromatic compounds and at 1624 and 1682 cm^− 1^ signals the C=C and C=O stretching of the carbonyl group^31^. A spectral peak witnessed at 1514 cm^− 1^ is linked to N–O stretching of nitrogen compound, whereas the 1219 cm^− 1^ signal concerns to C–O stretching of phenols and ester group^32^. A prominent spectral peak was detected at 771 cm^− 1^ implying C-H bending of aromatic ring structure^29^. Upon adsorption of 2,4-D, shifts in several key infrared signals were observed. The peaks at 1219, 1514, 3525, and 3730 cm^− 1^ shifted to 1213, 1517, 3562, and 3729 cm^− 1^, respectively. These changes suggest interactions between the C–O, N–O, and O–H functional groups and 2,4-D upon adsorption. Furthermore, a decrease in peak intensity at 771 and 1219 cm^− 1^, coupled with the vanishing of the peak at 1989 cm^− 1^, points to strong interactions between the C–H and C–O functional groups of 2,4-D and the TAAC.

**Fig. 4:**
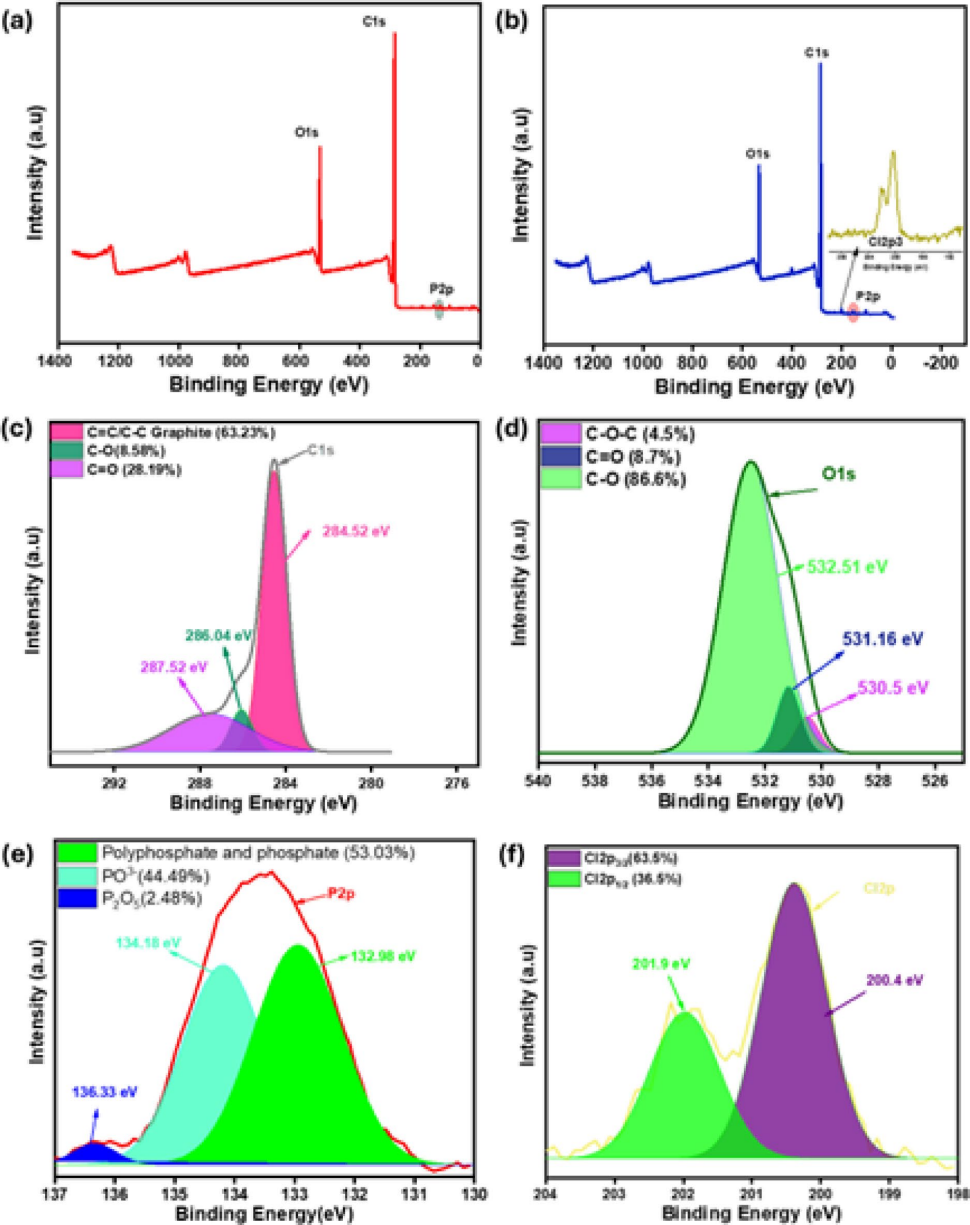
XPS spectra (**a**) pre adsorption (**b**) post adsorption. High resolution deconvolution post adsorption XPS spectra of (**c**) C1s (**d**) O1s (**e**) P2p (**f**) Cl2p.

Figure 4a shows the X-ray photoelectron spectroscopy (XPS) spectrum of the TAAC surface before adsorption, revealing its functional group composition. A dominant peak at 285 eV corresponds to C1s, while a signal at 533 eV indicates oxygen (O1s). The subtle signal at 134 eV corresponds to the P2p peak, attributed to the activation of TAAC with H_3_PO_4_^33^. Elemental analysis revealed the following atomic percentages: C1s (79.84%), O1s (17.09%), and P2p (0.61%). The results indicate that the TAAC derived from *T. aurea* leaves is predominantly carbonaceous, suggesting successful carbon enrichment. Oxygen is the next most abundant element. The occurrence and nature of these oxygen-containing functional groups are crucial, as they significantly influence the performance of the TAAC as an adsorbent^31^. Following adsorption, the elemental composition of TAAC changed to 78.94% carbon (C1s), and 17.37% oxygen (O1s), with only trace amounts of phosphorus (P2p). Specifically, the presence of chlorine (Cl2p) at 200 eV with a 0.96% in the post-adsorption XPS spectrum (Fig. 4b) authenticates the successful 2,4-D adsorption onto the TAAC. The changes observed in carbon and oxygen content suggest that these functional groups actively facilitated the process of 2,4-D adsorption. This observation is coherent with the results obtained from EDS analysis.

To investigate the specific functional groups involved in 2,4-D adsorption, high-resolution deconvolution of the post-adsorption XPS spectra was performed. The deconvoluted C1s spectrum (Fig. 4c) revealed three primary component constituents: C–C at 284.52 eV (63.23%), C–O bond observed at 286.04 eV (8.58%), and C=O signal with a binding energy of 287.52 eV (28.19%). The increased presence of C=O and C–O groups after 2,4-D adsorption, principally resulting from the carboxyl moieties within 2,4-D, suggests physisorption, likely through hydrogen bonding and pi-pi associations among the TAAC and 2,4-D^34^. This was also evident from the high-resolution O1s spectra (Fig. 4d) which revealed the occurrence of C–O at 532.51 eV (86.6%) and C=O at 531.16 eV (8.7%). Deconvolution of the P2p spectrum (Fig. 4e) revealed predominantly polyphosphate and phosphate groups at 132.98 eV (53.03%), along with metaphosphate ions at 134.18 eV (44.49%) and a minor presence of diphosphorus pentoxide at 136.33 eV^31^. The Cl2p spectrum (Fig. 4f) confirmed the presence of Cl2p_3/2_ at 200.4 eV (63.5%) and Cl2p_1/2_ at 201.9 eV (36.5%), indicative of organic chlorine. Similar results were obtained for Cl2p_3/2_ at 200.5 eV and Cl2p_1/2_ at 202.2 eV synthesized carbon nanofiber for 2,4-D removal^35^ and Cl2p_3/2_ at 200.6 eV and Cl2p_1/2_ at 202.2 eV for photochemical chlorinated graphene^36^. Collectively, the XPS analysis suggests that 2,4-D adsorption onto TAAC occurs via physisorption, involving both pi-pi stacking and hydrogen bonding.

### Adsorption studies

#### Impact of pH

**Fig. 5:**
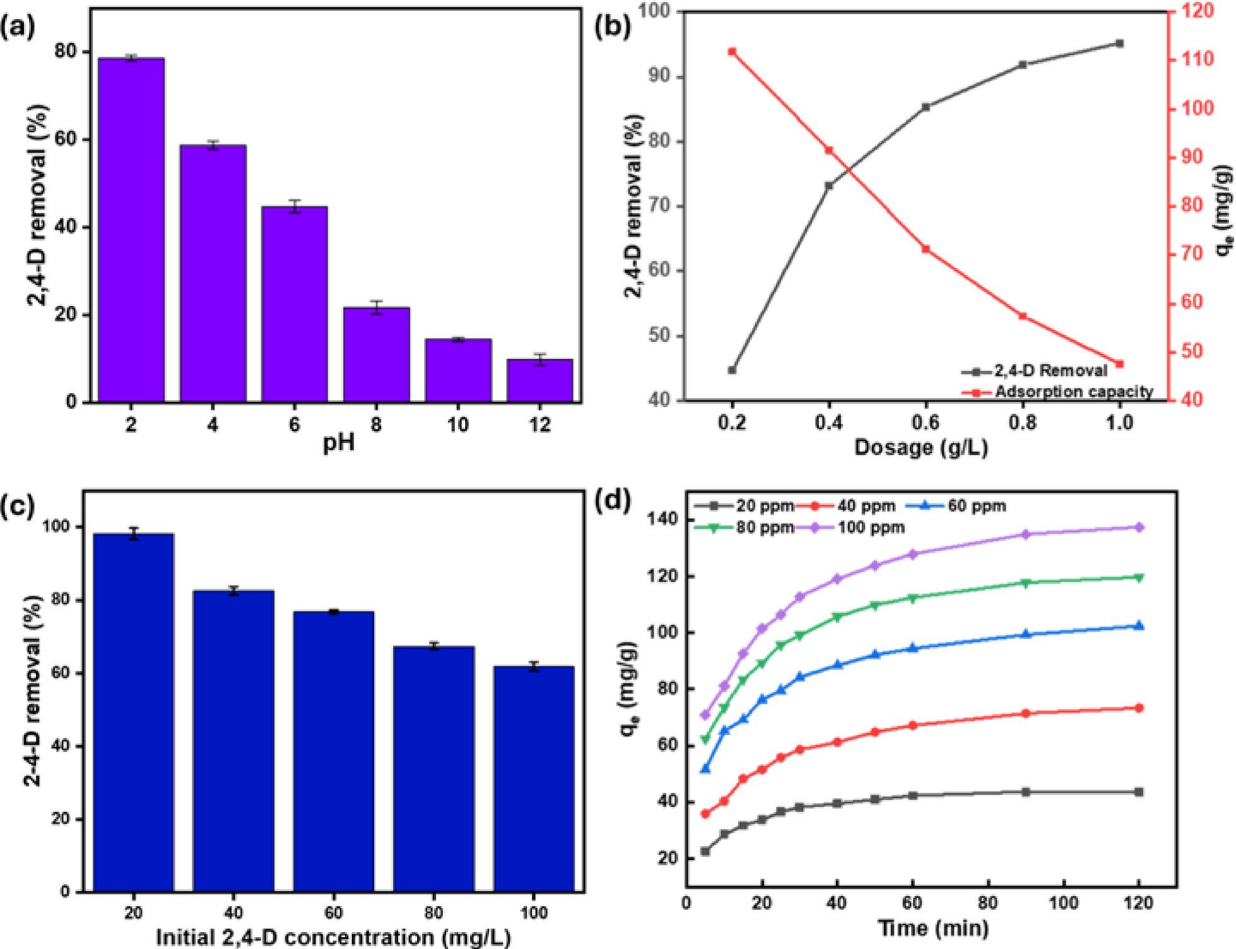
(**a**) Influence of pH (**b**) TAAC dosage (**c**) Initial 2,4-D concentration (**d**) contact time.

The solution pH was changed from 2 to 12 to assess the effect of pH on 2,4-D removal. The experiment commenced with 50 mg/L 2,4-D, accompanied by a TAAC dosage of 0.5 g/L. Figure 5a indicates that the high 2,4-D removal occurred with pH 2, aligning with previously documented research on 2,4-D removal^25^. The TAAC surface is positively charged below the pH_zpc_ (2.23). Hence, the electrostatic force of attraction may be engaged in the 2,4-D removal^7^. In contrast, the solution’s pH surpassing 2 leads to a reduction in the 2,4-D removal. This happened as pH levels exceeded 2.8 (pK_a_), leading to a significant fraction of the 2,4-D converting into an anionic form, which caused electrostatic repulsion^37^. Above pH 8, deprotonation of TAAC’s phenolic groups increases its negative surface charge, causing electrostatic repulsion between TAAC phenolate anions and 2,4-D anions, thus reducing 2,4-D removal^38^. Furthermore, at higher pH, hydroxyl ions compete with anionic 2,4-D for vacant sites on the TAAC^39^. These combined effects contribute to the observed reduction in 2,4-D removal as the pH increases.

#### Impact of dosage

The exploration aimed at the effects of dosage by changing the TAAC dose at incremental values between 0.2 and 1 g/L with constant initial 2,4-D concentration of 50 mg/L, pH at 2.0, temperature at 303 K, and agitation speed at 150 rpm. The data presented in Fig. 5b indicate a clear trend where the 2,4-D removal performance advances from 44.7 to 97.17% as the dosage increases. The adsorption capacity exhibits a drop from 111.75 to 47.58 mg/g as an increase in TAAC dosage. An increased TAAC dose resulted in an increased percentage of 2,4-D removal because of the enhanced quantity of active sites. However, increasing the TAAC dosage decreased the adsorption potential due to unused sites. A similar trend was observed in canola stalk-derived activated carbon^24^. The curves intersected at 0.44 g/L with a removal of about 75.65% and the adsorption potential of 88 mg/g, indicating that this dosage offered moderate results for both adsorption potential and percentage removal. As a result, this value was rounded off to 0.45 g/L and was opted for the succeeding tests.

#### Impact of 2,4-D concentration and contact time

The implications of the initial 2,4-D concentration and contact time were studied by altering the 2,4-D levels between 20 and 100 mg/L while keeping the pH at 2.0, TAAC dosage at 0.45 g/L, temperature at 303 K, and agitation speed at 150 rpm. Because of the abundance of active sites, adsorption was rapid at first ascribed to a high SSA coupled with a considerable pore volume^40^. However, as the adsorption sites became limited, uptake decreased, reaching equilibrium at 120 min, as demonstrated in (Fig. 5d). A swift initial depletion transitioned into a gradual deceleration is attributed to the gradual occupation of accessible sites on the particle surface^7^. Consequently, further studies were conducted using 120 min as the optimum contact time. A significant increase in adsorption potential, from 43.65 to 137.52 mg/g, was observed as the concentration changed from 20 to 100 mg/L. At higher concentrations, active site saturation, a steeper concentration gradient, and enhanced 2,4-D-TAAC interactions likely contribute to this increased capacity^41^. However, removal efficiency diminished from 98.22 to 61.88% over the same concentration range (Fig. 5c), possibly due to a reduction in accessible active sites. This demonstrates a strong dependence of 2,4-D adsorption on its initial concentration.

#### Adsorption kinetic, isotherm, and thermodynamics studies

**Fig. 6:**
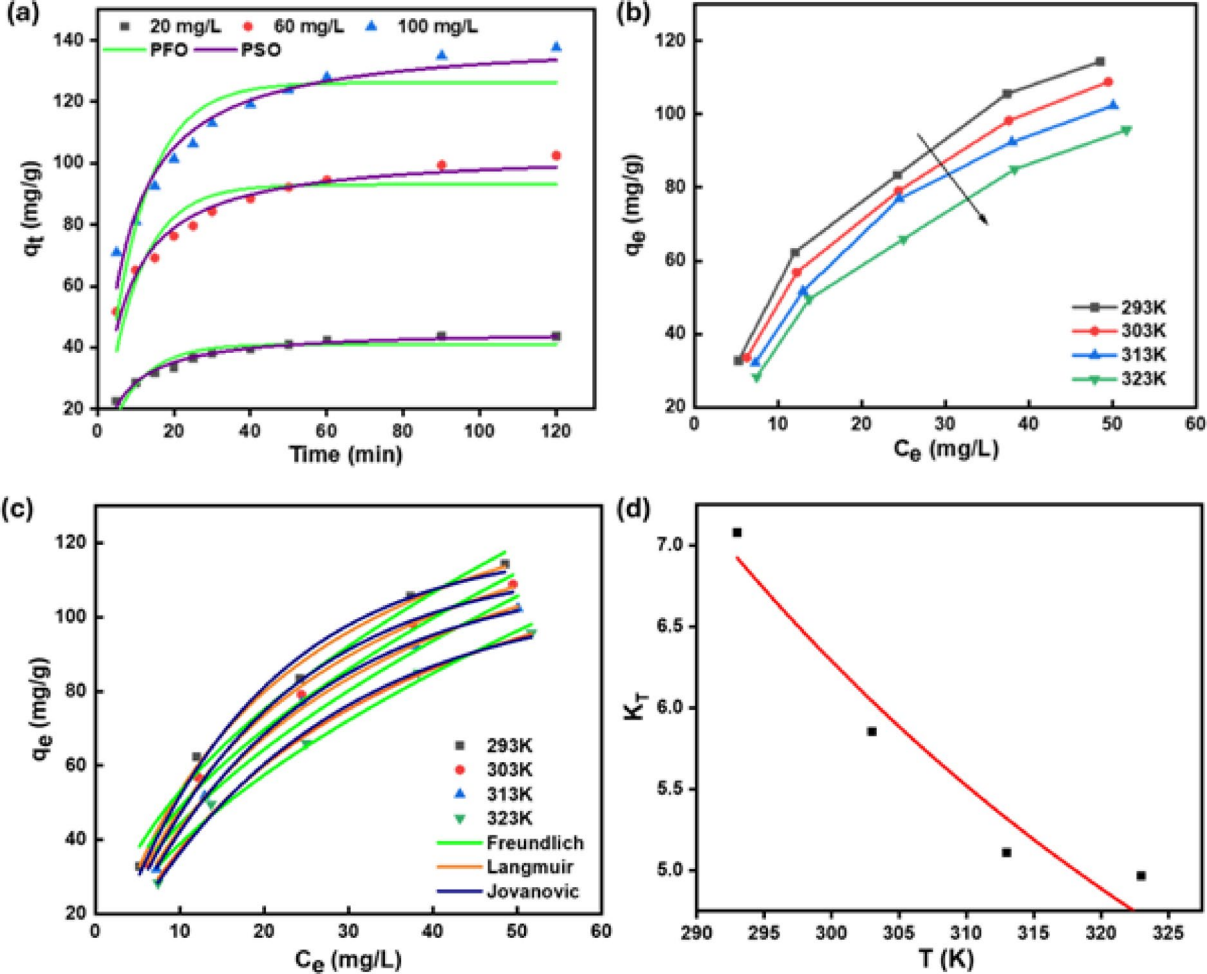
(**a**) Kinetic models (**b**) effect of temperature (**c**) isotherm models (**d**) thermodynamic plot for 2,4-D removal using TAAC.

The investigation into the kinetics of 2,4-D removal involved conducting adsorption studies under optimal conditions. Established kinetic model equations were used to analyze the experimental dataset, including PFO and PSO, through nonlinear regression analysis, as demonstrated in (Fig. 6a). The goodness-of-fit was assessed with R² and χ² values providing further confirmation of model suitability. Table 1 presents the assessed kinetic parameters at various 2,4-D concentrations. The PFO, PSO models exhibited R^2^ values of 0.809 and 0.962 accompanied by χ² values of 51.54, 10.24, respectively at 60 mg/L of 2,4-D concentration. Similar trends were observed for 20 and 100 mg/L of 2,4-D concentration as well. Additionally, the strong alignment between the experimental q_e, exp_ (102.46 mg/g) and the calculated q_e_ (103.94 mg/g) supports the reliability of the PSO model, corroborating that the 2,4-D removal adheres to PSO kinetics and indicating potent attraction between TAAC and 2,4-D. The strong agreement between experimental data and the PSO model indicates that chemisorption is the dominant process, with a rate-limiting step involving valence forces through electron sharing or exchange between the adsorbate and the adsorbent’s active sites. This interpretation is further supported by our FTIR and XPS analyses, which reveal the involvement of –OH, C=O, and C–O functional groups on the TAAC surface in specific interactions with 2,4-D molecules. Consequently, the strong correlation with the PSO model reinforces the conclusion that chemical interactions, such as hydrogen bonding, π–π interactions, and electron donor–acceptor mechanisms, primarily govern the adsorption process. These findings correspond with previous research regarding the removal of 2,4-D utilizing polydopamine/polyacrylamide co-deposited magnetic Sporopollenin^42^, La/Rb bimetal-organic frameworks^43^ and MIL-88(Fe)-NH_2_^44^.

**Table 1:** Estimated parameters for the kinetic model for the adsorption of 2,4-D on TAAC.

2,4-D concentration (mg/L)			
Kinetic model	20	60	100
PFO			
$\:{\mathbf{k}}_{1}\:\left({\mathbf{m}\mathbf{i}\mathbf{n}}^{-1}\right)$	0.112	0.108	0.099
$\:{\mathbf{q}}_{\mathbf{e}}\:(\mathbf{m}\mathbf{g}/\mathbf{g})$	41.15	93.05	126.22
$\:{\mathbf{R}}^{2}$	0.868	0.809	0.807
χ^2^	6.64	51.54	101.75
PSO			
$\:{\mathbf{k}}_{2}\:(\mathbf{g}/\:\mathbf{m}\mathbf{g}.\mathbf{m}\mathbf{i}\mathbf{n})$	0.0037	0.0015	0.001
$\:{\mathbf{q}}_{\mathbf{e}}\:\left(\mathbf{m}\mathbf{g}/\mathbf{g}\right)$	45.72	103.94	141.25
$\:{\mathbf{q}}_{\varvec{e},\varvec{e}\varvec{x}\varvec{p}}\:\left(\mathbf{m}\mathbf{g}/\mathbf{g}\right)$	43.65	102.46	137.52
$\:{\mathbf{R}}^{2}$	0.983	0.962	0.952
χ^2^	0.85	10.24	25.06

Figure 6b depicts the impact of temperature on 2,4-D removal by TAAC at varied concentrations. The findings uncover that the adsorption capacity of TAAC for 2,4-D declines as the temperature ascents from 293 to 323 K. This decline can be accredited to the greater kinetic energy of adsorbed molecules at elevated temperatures, which enhances the probability of desorption from the activated carbon surface that results in a reduced adsorption performance. Higher temperatures may cause structural changes in the activated carbon, such as an enlarged pore, which can indicate a decline in both surface area and adsorption efficiency. Hence, the stated fall could be due to a lower intensity of available adsorption sites or a decline in the formation of adsorption layers.

This study involved an examination of three conventional adsorption isotherms: Langmuir, Freundlich, and Jovanovic at a temperature range between 293 and 313 K. Figure 6c illustrates the non-linear plot of these isotherms, while Table 2 contains the corresponding fitting parameters. The Langmuir model demonstrated a significant relationship with the adsorption of 2,4-D on TAAC. The observation was made that the maximum uptake exhibits an increase as the temperature decreases, ranging from 152.75 to 161.70 mg/g. Many studies have reported a similar trend of increased adsorption potential with temperature, including wheat husk^45^ and *Physalis peruviana* fruit treated with H_2_SO_4_^46^ for 2,4-D removal. Furthermore, the n, Freundlich constant were in the range of 1.96 to 1.78, falls between 1 and 10, and the Langmuir separation factor, R_L_ (1/(1 + K_L_*C_o_) ) ranges between 0.29 and 0.38, lies within 0 to 1 for all the temperature range, providing evidence for the effective adsorption of 2,4-D onto TAAC^47^.

**Table 2:** Fitted values of isotherm parameters for 2,4-D removal using TAAC.

Temperature (K)				
Model	293	303	313	323
Langmuir				
$\:{\mathbf{q}}_{\mathbf{m}}$ (mg/g)	161.70	158.28	157.77	152.75
$\:{\mathbf{K}}_{\mathbf{L}}\:$(L/mg)	0.0487	0.0435	0.0373	0.0323
$\:{\mathbf{R}}^{2}$	0.994	0.997	0.998	0.995
χ^2^	8.86	3.39	1.69	4.62
Freundlich				
$\:{\mathbf{K}}_{\mathbf{F}}$ ((mg/g) (mg/L)^− 1/n^)	16.54	14.76	12.70	10.59
$\:\frac{1}{\mathbf{n}}$ (dimensionless)	0.51	0.52	0.54	0.56
$\:{\mathbf{R}}^{2}$	0.985	0.988	0.980	0.985
χ^2^	21.79	13.96	22.07	13.83
Jovanovic				
$\:{\varvec{q}}_{\varvec{m}}\:$(mg/g)	119.99	115.63	112.44	107.33
$\:{\varvec{K}}_{\varvec{j}}\:$(L/mg)	0.056	0.052	0.047	0.041
$\:{\mathbf{R}}^{2}$	0.987	0.992	0.999	0.993
χ^2^	18.73	9.87	0.67	6.85

The R^2^ values for Langmuir, Jovanovic, and Freundlich were 0.997, 0.992, and 0.988, while the χ^2^ values were 3.39, 9.87, and 13.96, respectively, at 303 K. The analysis of the highest R^2^ and the lowest χ^2^ value at various temperature ranges indicates that the Langmuir isotherm model is highest applicable for the removal of 2,4-D using TAAC, suggesting monolayer adsorption on the uniform adsorption sites of TAAC^48^. The 2,4-D removal values described in the literature (Table 3) are lower than the maximum uptake of 158.28 mg/g accomplished by TAAC at 303 K.

**Table 3:** Evaluation of 2,4-D removal using several forms of activated carbon-based adsorbent.

Activated carbon	SSA (m^2^/g)	pH	Dose (g/L)	T (K)	Time (min)	2,4-D (mg/L)	Adsorption potential (mg/g)	Ref
Activated carbon derived from polyaniline	–	–	0.025	298	30	50	34.48	^52^
Date palm coir waste derived nano-activated carbon	947	2	3	303	90	100	50.25	^8^
Magnetic activated carbon derived from *Ulva prolifera*	292.51	2	2	303	60	100	60.61	^53^
*RitamaMonosperma* (L.) Boiss derived activated carbon with H_3_PO_4_ activation	–	2	0.8	298	60	10	69.44	^6^
*Balanites aegyptiaca* seed shell derived activated carbon	417	2	0.8	308	250	80	69.93	^54^
Activated carbon derived from date stone	659	3	0.6	298	360	90	76.52	^55^
Kraft lignin derived N-doped activated carbon	1000	3	0.8	298	600	80	87 ± 3	^56^
Composite of activated carbon/graphene oxide from *Thysanolaena maxima* biomass	960	2	0.1	298	120	150	98.469	^40^
Canola stalk-derived activated carbon	556.78	2	0.3	298	60	150	135.8	^24^
Aminosilane derived activated carbon	834	3	0.4	298	120	6.25–150	143	^57^
*T. aurea* leaf-based activated carbon with H_3_PO_4_ activation	773.21	2	0.45	303	120	50	158.28	This study

The possibility of 2,4-D adsorption on TAAC was validated by thermodynamic tests. Thermodynamic parameters, ΔG°, ΔH°, and ΔS°, were established utilizing the Van’t Hoff plot, as seen in (Fig. 6d). These findings demonstrated the thermodynamic spontaneity and feasibility of the adsorption as evidenced by the negative ΔG° values at all evaluated temperatures (Table 4). This highlights the adaptability of the TAAC over a wide temperature range. The negative enthalpy value (ΔH° = −10.04 kJ/mol) demonstrates that the adsorption is exothermic, with a range between 0.4 and 80 kJ/mol, characteristic of physisorption^49^. Additionally, the negative entropy value (ΔS° = −18.198 J/mol K) represents a decline in randomness at the solid-solution interface when 2,4-D is adsorbed onto the active sites of TAAC^39^.

**Table 4:** Thermodynamic parameters for the removal of 2,4-D by TAAC.

Thermodynamic model					
T (K)	ΔG° (kJ/mol)	ΔH° (kJ/mol)	ΔS° (J/mol K)	R^2^	χ^2^
293	–4.76	–10.04	–18.198	0.941	0.081
303	–4.45				
313	–4.24				
323	–4.13				

#### Adsorption mechanism

**Fig. 7:**
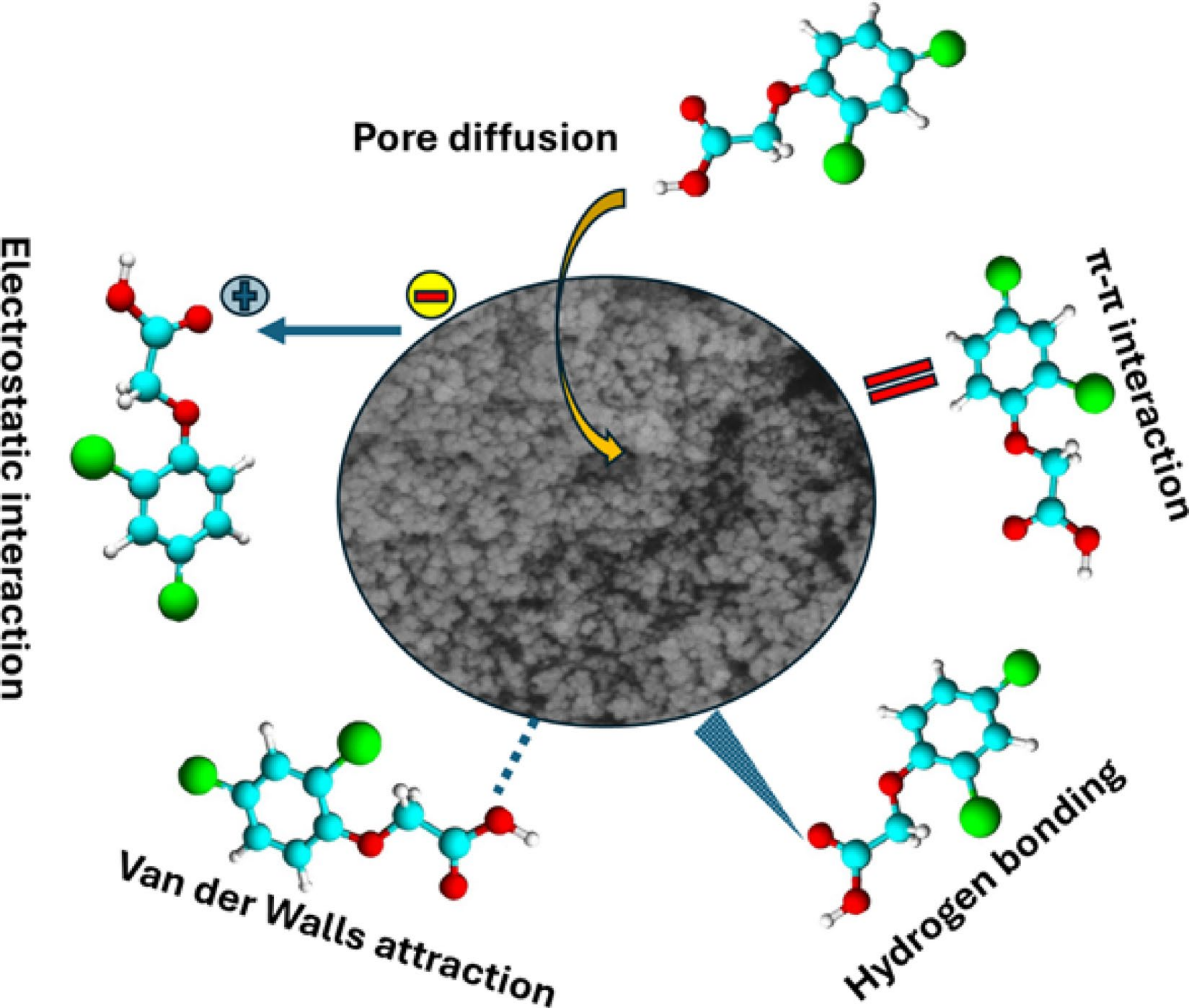
Proposed mechanism for the adsorption of 2,4-D on TAAC.

Considering the preceding discussions, a visual model of the hypothesized 2,4-D adsorption mechanism onto TAAC is shown (Fig. 7). Post-adsorption pore filling was observed in FESEM images. BET analysis confirmed the mesoporous properties of TAAC, with a pore diameter of 3.99 nm. EDS spectra of after-adsorption showed an increased Cl intensity, indicating a 2.4-D uptake. Furthermore, 2,4-D adsorption altered the carbon structure, shifting the (002) plane peak in XRD analysis. This shift suggests changes in interlayer spacing and reduced graphitic content due to 2,4-D binding. FTIR analysis suggested that this interaction involves hydrogen bonding and π-π interactions, where electrons are exchanged between aromatic rings. Post adsorption FTIR spectra showed shifts in the peaks at 1219, 1514, and 3730 cm⁻¹. Moreover, the occurrence of the C–C group also confirmed at 1624 cm⁻¹ indicates potential π-π interaction with 2,4-D aromatic ring. Oxygen-containing moieties, including –OH, and C=O, can donate electrons in π-π interaction concerning the aromatic ring of adsorbed 2,4-D molecules, which function as electron acceptors. The C–H aromatic ring band at 771 cm⁻¹ suggests similar interactions. Consequently, π-π stacking between 2,4-D and TAAC is another possibility with the strength of these interactions influenced by the number of aromatic rings and hydrophobicity^25^. Furthermore, XPS analysis shows increased C–O and C=O groups on TAAC’s surface after 2,4-D removal, attributable to 2,4-D carboxyl groups. This suggests physisorption, possibly involving intermolecular forces including π-π interaction and hydrogen bonding among TAAC and 2,4-D^25^, in agreement with FTIR analysis. The presence of chlorine in 2,4-D explains the existence of a Cl2p peak after adsorption. Deconvolution of the Cl2p spectra revealed a Cl2p_3/2_ at 200.4 eV and Cl2p_1/2_ at 201.9 eV characteristic of organic chlorine^35^. At pH 2, electrostatic attraction is the primary adsorption mechanism on TAAC, though Van der Waals forces and hydrogen bonding play a role as well. Considering the thermodynamic investigations, physisorption is the main process (ΔH° = −10.04 kJ/mol). Therefore, multiple processes, including pore filling, hydrogen bonding, electrostatic and Van der Waals forces, and π-π interactions are engaged in the adsorption process, which supports the multi-mechanistic character of 2,4-D adsorption on TAAC.

#### Regeneration potential

**Fig. 8:**
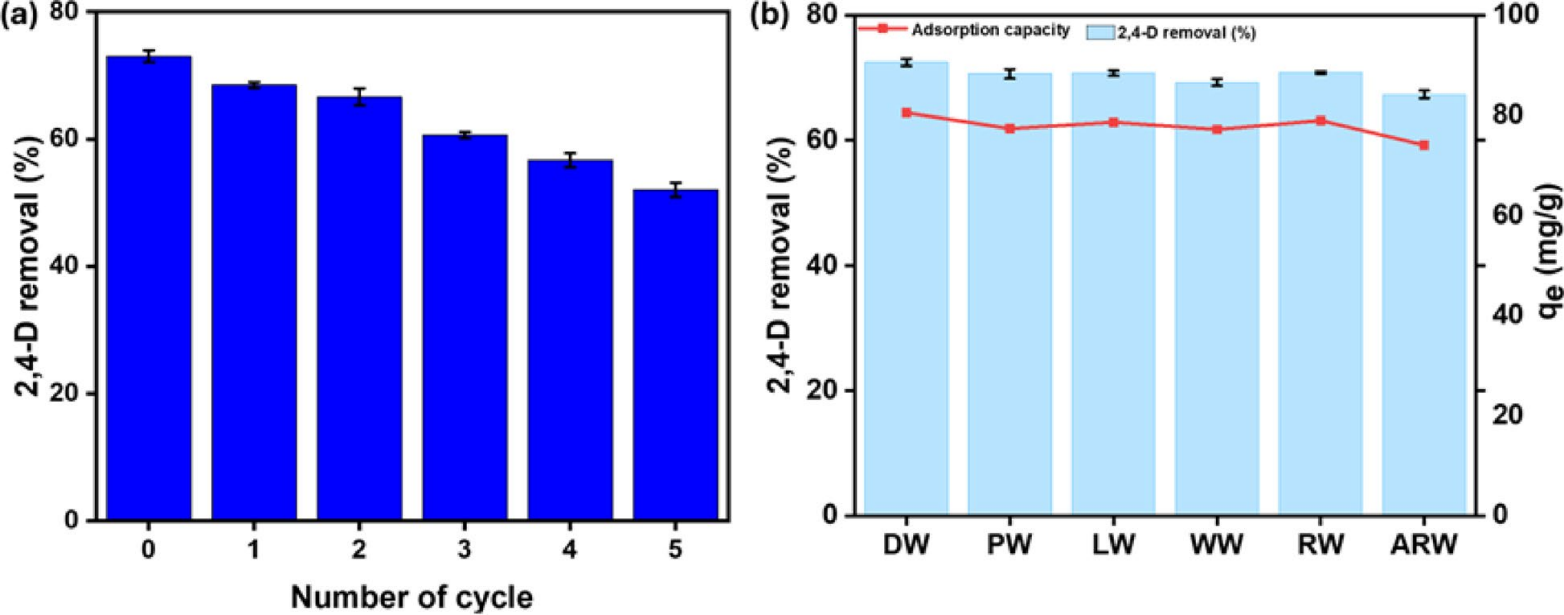
(**a**) Regeneration potential of TAAC (**b**) Spiked 2,4-D studies for various water sources.

For large-scale applications, spent adsorbent regeneration is crucial because it allows adsorbents to be reused across many cycles, which reduces operating costs. 0.1 M NaOH was used as the regeneration eluent in this investigation. Because of the adsorption of OH⁻ ions, the basic environment made it easier for 2,4-D to deprotonate. Furthermore, the anionic 2,4-D molecules were rejected by the negative charges that the high pH created on the TAAC surface. The regeneration capability of 0.1 M NaOH is illustrated in (Fig. 8a), evidencing a decrement in 2,4-D removal from 72.48 to 52.02% after five cycles. The slight fluctuation in removal efficiency throughout the cycles indicated the robust regeneration capability of TAAC. Comparable findings were recorded using date palm leaves for 2,4-D removal^50^.

#### Assessment of 2,4-D removal from different water sources

Water samples were collected from different sources, including ponds (PW), lakes (LW), wells (WW), rivers (RW), and agricultural runoff (ARW) near the Manipal campus. The removal efficiency of TAAC for 2,4-D in these spiked samples is shown in Fig. 8b compared with distilled water (DW). Experiments were performed under optimal conditions determined from batch studies. No notable differences in adsorption capacity were observed across the tested water samples compared to distilled water, demonstrating that the presence of different constituents in the water samples did not hinder the adsorption capacity. These results demonstrate the efficacy of TAAC for removing 2,4-D from diverse water matrices.

#### Impact of coexisting ions

**Fig. 9:**
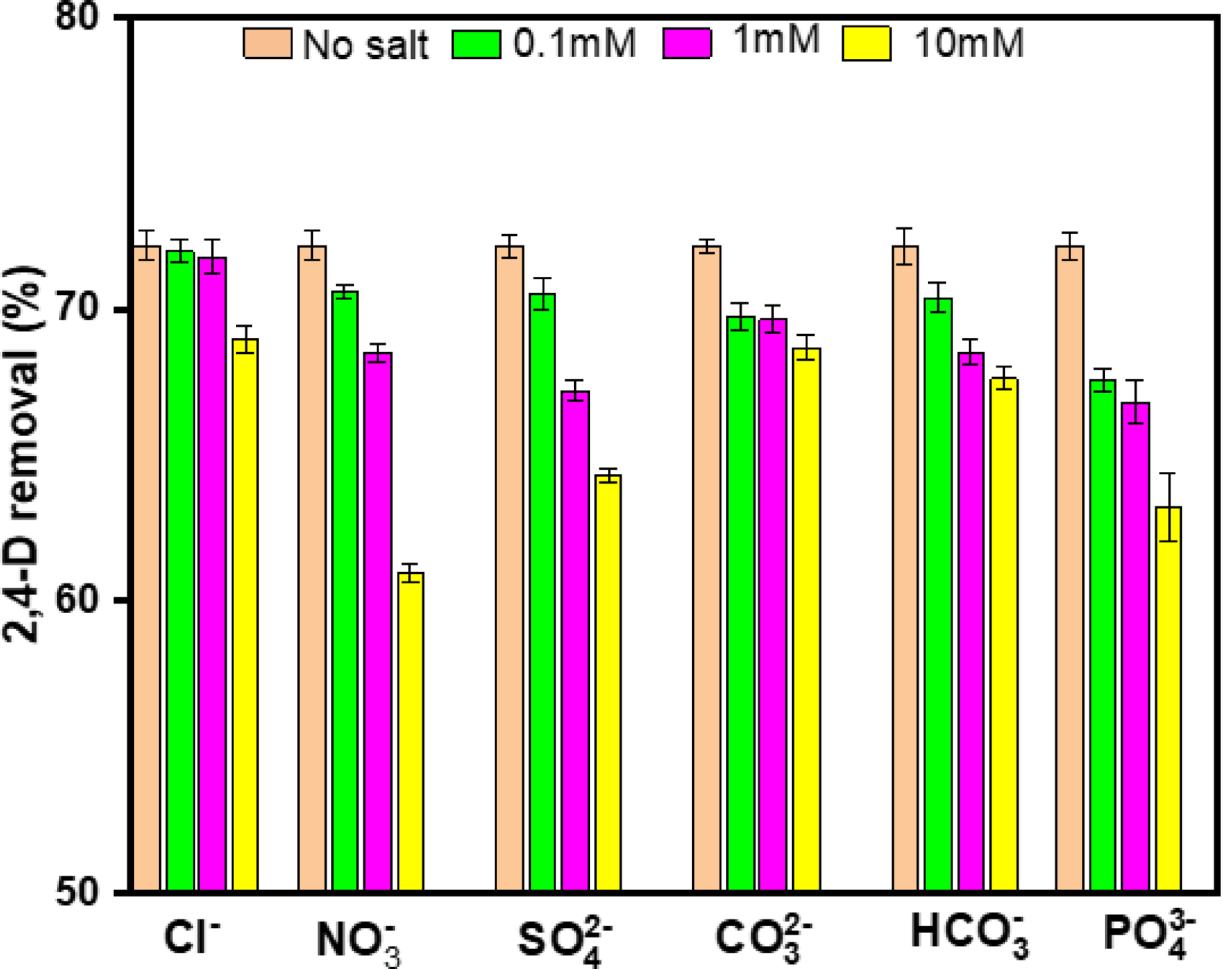
Impact of inorganic ions on 2,4-D removal using TAAC.

Wastewater often includes a wide variety of inorganic ions; thus, it is essential to examine the impact of these ions on the effectiveness of 2,4-D adsorption in aqueous water solutions. The ionic concentrations used in this investigation ranged from 0.1 to 10 mM, and the optimal conditions were used for the study (Fig. 9). This behavior was seen in the presence of Cl⁻, NO₃⁻, SO₄²⁻, CO₃²⁻, HCO₃⁻, and PO₄³⁻ ions. However, the minimal strengths of 0.1 mM and 1 mM had less impact, and a significant reduction in adsorption efficiency was observed at 10 mM^51^. The order of impact from coexisting ions on 2,4-D adsorption was Cl⁻ > CO₃²⁻ > HCO₃⁻ > SO₄²⁻ > PO₄³⁻ > NO₃⁻. These findings suggest that nitrate ions at high concentrations have a strong affinity for TAAC, and their presence in wastewater may notably reduce TAAC’s efficiency in removing 2,4-D.

## Conclusions

A sustainable mesoporous activated carbon was synthesized from *T. aurea* leaves using H_3_PO_4_ activation. Characterization studies, including XRD analysis, confirmed the amorphous nature of TAAC, and elemental analysis confirmed successful 2,4-D uptake onto the TAAC surface. FTIR and XPS analyses revealed the involvement of C-O, O-H, and C-H functional groups in 2,4-D adsorption. The kinetic data was precisely depicted by a pseudo-second-order model and isotherm data fitted satisfactorily to the Langmuir isotherm model. The 2,4-D adsorption was shown to be exothermic and spontaneous, according to the thermodynamic study. Characterization and adsorption studies collectively indicate that the adsorption process involves multiple mechanisms, including pore filling, hydrogen bonding, electrostatic attractions, Van der Waals forces, and pi-pi interaction, thus supporting the multi-mechanistic nature of 2,4-D adsorption on TAAC. Following five cycles of processing, the TAAC showed promising regeneration capability. The presence of higher concentrations of inorganic ions, including phosphates and nitrates, strongly impacted the adsorption efficiency of 2,4-D. However, no notable differences in adsorption capacity were observed across the tested water samples, suggesting that TAAC is highly effective for eliminating 2,4-D from both water and wastewater.

## Data Availability

The authors declare that the data supporting the findings of this study are available within the article.

## Declarations

### Plant guidelines

The authors confirm that the use of plant/plant parts in the present study complies with international, national and/or institutional guidelines.

### Source of the plant used in your study

All plant name and the source of them are in the Methods section.
